# Complete mitochondrial genome of the freshwater prawn *Palaemon capensis* (Crustacea: Palaemonidae)

**DOI:** 10.1080/23802359.2017.1390400

**Published:** 2017-10-17

**Authors:** Louisa E. Wood, Sammy De Grave, Carel J. van Heerden, Savel R. Daniels

**Affiliations:** a Department of Botany and Zoology, University of Stellenbosch, Stellenbosch, South Africa;; b Oxford University Museum of Natural History, Oxford, UK;; c Central Analytical Facility (CAF), DNA Sequencing Unit, Stellenbosch University, Stellenbosch, South Africa

**Keywords:** Ion torrent, mitogenome, phylogeny, *Palaemon*

## Abstract

The complete mitogenome of *Palaemon capensis* is presented here. The mitogenome is 15,925 bp in length and comprises 13 protein coding genes, 2 ribosomal subunit genes, 22 transfer RNAs, and a non-coding AT-rich region. The PCGs were used to perform a phylogenetic analysis together with other Caridea representatives with mitogenome data from GenBank, placing *P. capensis* sister to a clade comprising *P. serenus*, *P. gravieri*, and *P. carinicauda* in the family Palaemonidae.

*Palaemon capensis* De Man, 1897 is a freshwater shrimp species that is endemic to South Africa (Coetzee 1988). It is the only known native freshwater prawn species to occur in the temperate Cape Floristic Region of South Africa (Wood et al. 2017), where its survival has been shown to be influenced by recent human-mediated modifications to their riverine habitat (Coetzee 1991). Further, the systematic position of this species within the genus *Palaemon* remains unknown, as it was not included in the phylogenetic studies of Ashelby et al. (2012) and Carvalho et al. (2017). To provide additional molecular resources for future conservation and systematic based studies, the mitogenome of *P. capensis* is described herein using the Ion Torrent platform.

The prawn specimen was collected from the Breede River (−34.0675, 20.4138) on 5th July 2015 and vouchered in the collections of the South African Museum (SAMC-A088831, IZIKO Museums of Cape Town, South Africa). Total genomic DNA was extracted from the abdominal muscle using a Nucleospin kit (Macherey-Nagel, Düren, Germany). The complete mitogenome was determined using the Ion Torrent PGM^™^ sequencer (Life Technologies, Carlsbad, CA) at the Central Analytical Facility, Stellenbosch University. PCGs and *rRNA* genes were annotated by MITOS (Bernt et al. 2013) and *tRNA* genes were determined by both MITOS and tRNAscan- SE version 1.21 (Library of Medicine, Bethesda, MD). A complete mitogenome of 15,925 bp was obtained (36.2% A, 32.9% T, 11.5% G, and 19.4% C).

**Figure 1.**
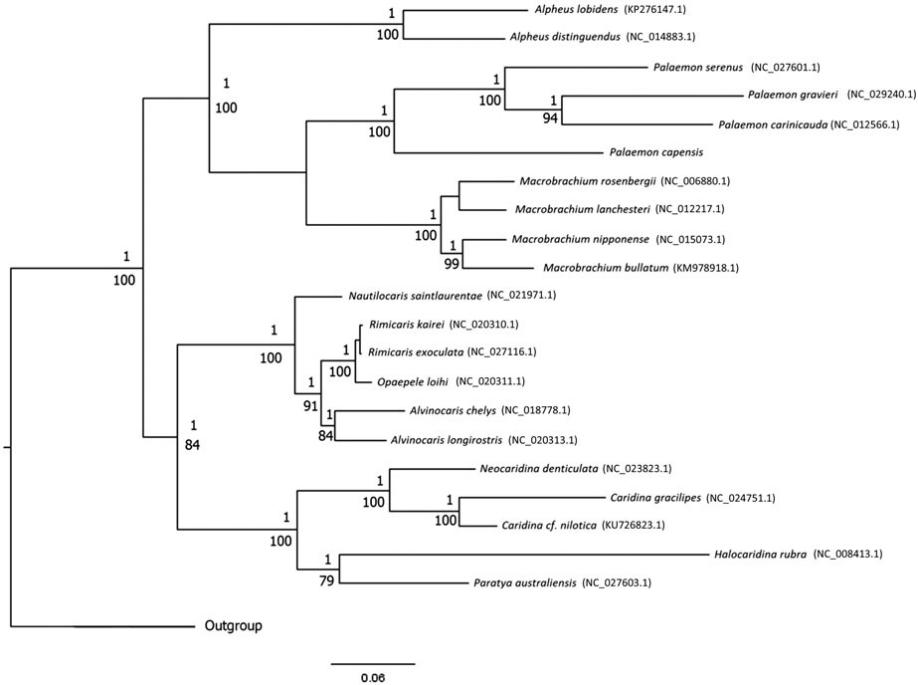
Phylogenetic tree inferred from the concatenated amino acid (AA) sequences of 13 PCGs (3534 AAs) of 21 Caridea species and two Dendrobranchiata outgroups highlighting the phylogenetic position of *P. capensis*. The Bayesian inference topology is shown with nodes displaying BI posterior probabilities and ML bootstrap values. Sequences were aligned using MUSCLE version 3.8.31 (Edgar 2004) and trimmed by Gblocks version 0.91b (Castresana 2000; Talavera and Castresana 2007), with 3534 concatenated amino acids retained (86%). PartitionFinder version 1.1.1 (Lanfear et al. 2012) was used to select the best partitioning scheme and evolutionary models.

The mitochondrial genome contained 13 PCGs, two *rRNA* genes, 22 *tRNA* genes, and a potential control region. Eight tRNAs, four protein coding genes, and the two *rRNA* genes were encoded on the light strand with the remainder encoded on the heavy strand. Allowing for overlap, protein-coding genes accounted for 70.00% of the genomic sequence; *rRNA* genes 13.64%, *tRN*A genes 9.02%, and non-coding DNA 7.34%. Comparison to the mitogenomes of *Palaemon serenus, P. gravieri,* and *P. carinicauda* (Shen et al. 2009; Kim et al. 2015; Gan et al. 2016) indicated that the gene arrangement of *P. capensis* is well conserved, from COX1 to tRNA^Tyr^. All these species exhibit a reversal of the T and P *tRNA* genes, thus deviating from the primitive pancrustacean mitogenome order (Miller et al. 2005). ATN was the start codon for all protein coding genes with the exception of COX1 in which ACG was used and NAD4 in which CAC was used. The lengths of the *LrRNA* and the *SrRNA* genes in *P. capensis* were 1371 and 801 bp, respectively. The *P. capensis* mitogenome had 15 small non-coding regions ranging from 1 to 38 bp. The only large non-coding region was 1085 bp in length, which is likely to be the control region (A + T content of 80.4%).

The reconstructed phylogeny supported a sister relationship to a clade comprising *P. serenus, P. gravieri*, and *P. carinicauda* within the *Palaemonidae* (Figure 1).

The mitochondrial genome of *P. capensis* was deposited in GenBank under the accession number MF797833.
